# Effects of Polymorphisms -1112C/T and +2044A/G in Interleukin-13 Gene on Asthma Risk: A Meta-Analysis

**DOI:** 10.1371/journal.pone.0056065

**Published:** 2013-02-20

**Authors:** Wei Nie, Yongan Liu, Jiarong Bian, Bin Li, Qingyu Xiu

**Affiliations:** 1 Department of Respiratory Disease, Shanghai Changzheng Hospital, Second Military Medical University, Shanghai, People’s Republic of China; 2 Department of Intensive Care Medicine, No. 411 Hospital of PLA, Shanghai, People’s Republic of China; 3 Department of Radiology, Shanghai Changzheng Hospital, Second Military Medical University, Shanghai, People’s Republic of China; INRS - Institut Armand Frappier, Canada

## Abstract

**Background:**

Associations between interleukin-13 (IL-13) polymorphisms and asthma risk remained controversial and ambiguous. Therefore, we performed a meta-analysis to assess the associations between *IL-13* polymorphisms and asthma susceptibility.

**Methods:**

Pubmed, EMBASE, Chinese National Knowledge Infrastructure (CNKI) and Wangfang databases were searched. Odds ratios (ORs) with 95% confidence intervals (CIs) were used to calculate the strength of association in the random-effects model.

**Results:**

Thirty-four studies were included in this meta-analysis. The results indicated that *IL13* -1112C/T polymorphism was significantly associated with asthma risk (OR = 1.20, 95% CI 1.08–1.34, *P* = 0.0009) in a dominant genetic model. When stratifying for race, *IL13* -1112C/T polymorphism exhibited increased asthma risk in Caucasians (OR = 1.30, 95% CI 1.09–1.55, *P = *0.003), while no significant association was found in Asians and African Americans. In the subgroup analysis based on atopic status, significant association was observed in atopic patients (OR = 1.25, 95% CI 1.07–1.45, *P = *0.004) but not in the non-atopic patients. In addition, a significant association between *IL13*+2044A/G polymorphism and asthma risk was observed (OR = 1.18, 95% CI 1.08–1.28, *P* = 0.0002). In the subgroup analysis by ethnicity, there were significant associations between *IL13*+2044A/G polymorphism and asthma risk in Asians (OR = 1.19, 95% CI 1.04–1.36, *P = *0.01) and Caucasians (OR = 1.22, 95% CI 1.06–1.40, *P = *0.005) but not in African Americans. In the subgroup analysis stratified by atopic status, a marginal significant association was found in atopic patients (OR = 1.12, 95% CI 1.00–1.26, *P = *0.05).

**Conclusions:**

This meta-analysis suggested that the *IL13* -1112C/T and +2044A/G polymorphisms were risk factors for asthma.

## Introduction

Asthma is one of the most common chronic respiratory diseases, characterized by wheezing, cough, and bronchial hyperresponsiveness. It is believed to be a multifactorial disorder with a strong genetic component [1], [2]. Interleukin-13 (IL-13) is a central effector cytokine of allergic inflammation. Huang et al. [3] found that a significant enhancement of both IL-13 transcripts and secreted proteins in the allergen-challenged bronchoalveolar lavage (BAL) compared with the saline-challenged control sites of asthmatic and rhinitic patients. Furthermore, in human subjects with asthma, the IL-13 concentration in peripheral blood was increased across disease severity in a stable state and was up-regulated at exacerbations [4], [5]. Recently, Corren et al. [6] reported that lebrikizumab treatment in 219 adults who had inadequately controlled asthma was associated with improved lung function. These results strongly suggested that IL-13 had an important role in the pathogenesis of asthma and the *IL-13* gene may be a susceptibility gene of asthma.

So far, a lot of studies investigated the association between the *IL-13* gene polymorphisms and susceptibility of asthma [7]–[40]. Most of them focused on two polymorphisms: -1112C/T and +2044A/G. However, the results from these studies were inconsistent. Although two meta-analyses on these polymorphisms have be published [41], [42], some inconsistent results still existed. For example, Yang et al. [41] reported that *IL-13*+2044A allele was associated with an increased risk of asthma among Asians but not among Caucasians. However, Cui et al. [42] found that *IL-13*+2044A/G polymorphism was associated Caucasians but not Asians. In addition, these two meta-analyses did not evaluate the association between *IL-13* polymorphisms and atopic asthma. Hence, we performed a meta-analysis of all eligible studies to derive more precise estimation of the associations of *IL-13* −1112C/T and +2044A/G polymorphisms with asthma risks. This was, to our knowledge, the most comprehensive meta-analysis of the association between *IL-13* polymorphisms and asthma susceptibility.

## Methods

### Publication Search

Published studies were identified through a computerized search of Pubmed, EMBASE, Chinese National Knowledge Infrastructure (CNKI) and Wangfang databases (Last search was updated on October, 2012). The search terms were used as follows: (asthma or asthmatic) and (interleukin-13 or IL-13) and (polymorphism or mutation or variant). We also perused the reference lists of all retrieved articles and relevant reviews. There was no language restriction.

### Inclusion and Exclusion Criteria

Studies included in the current meta-analysis should meet the following criteria: (1) evaluation of the polymorphisms in *IL-13* gene and asthma risk, (2) using a case-control design, (3) genotype distributions in both cases and controls should be available for estimating an odds ratio (OR) with 95% confidence interval (CI).

Studies were excluded if one of the following existed: (1) not relevant to *IL-13* or asthma risk, (2) not designed as case-control studies, (3) genotype frequencies or number not offered, (4) non-clinical studies, (5) editorials, reviews and abstracts, and (6) not consistent with Hardy-Weinberg equilibrium (HWE). In the case of overlapping studies, only the one with the largest sample numbers was included.

### Data Extraction

Data were extracted from all eligible studies independently by two of the authors (Nie and Liu). The relevant data were extracted into predesigned data collection forms. The following information was collected from each study: first author’s name, year of publication, original country, ethnicity, age group, atopic status, sample size, genotyping method, and genotype number in cases and controls. We verified accuracy of data by comparing collection forms from each investigator. If a decision could not be made regarding inclusion, the full text of the article was examined.

### Qualitative Assessment

Two authors (Nie and Liu) assessed the quality of each study independently. The quality scoring system was based on traditional epidemiologic considerations and asthma genetic issues [43]. Scores ranged from the lowest zero to the highest fifteen. Studies with quality scores ≤4 were defined as low quality studies [44].

### Statistical Analysis

When the data from at least 3 similar studies were available, meta-analysis was performed. The strength of the association between the *IL-13* polymorphisms and asthma risk was measured by ORs and 95% CIs. The statistical significance of summary OR was determined with *Z* test. OR1, OR2, and OR3 were calculated for the genotypes: 1). TT vs. CC (OR1), TC vs. CC (OR2), and TT vs. TC (OR3) for the -1112C/T polymorphism, 2). AA vs. GG (OR1), AG vs. GG (OR2), and AA vs. AG (OR3) for the +2044A/G polymorphism. These pairwise differences were used to indicate the most appropriate genetic model [45]–[47]. Once the best genetic model was identified, this model was used to collapse the three genotypes into two groups (except in the case of a codominant model) and to pool the results. We used a random-effects model to calculate the pooled ORs.

Heterogeneity among studies was examined with *I^2^* statistic. *I^2^* takes a value of 0–100% (*I^2^* = 0–25%, no heterogeneity; *I^2^* = 25–50%, moderate heterogeneity; *I^2^* = 50–75%, large heterogeneity; *I^2^* = 75–100%, extreme heterogeneity). A chi-square based Q-test was also performed to check the betweenstudy heterogeneity, which was considered to be significant for *P<*0.10. To explore the source of the heterogeneity and evaluate the ethnic-specific, atopic-specific effects, subgroup analyses were performed by ethnicity and atopic status. To access the stability of the meta-analysis, one-way sensitivity analyses were carried out. We did cumulative meta-analysis by undertaking sequential random-effects pooling, starting with the earliest studies. Results were presented as a series of mini meta-analyses, which were ordered chronologically in a forest plot to show the consequence of adding studies on the effect size. Departure from HWE in controls was tested by the chi-square test. Publication bias was assessed by visual inspection of funnel plots, in which the standard error of log (OR) of each study was plotted against its log (OR). Funnel plot asymmetry was assessed by Egger’s linear regression test [48].

All statistical tests were performed by using the Revman 5.1 software (Nordic Cochrane Center, Copenhagen, Denmark) and STATA 11.0 software (Stata Corporation, College Station, TX). A *P* value <0.05 was considered statistically significant.

## Results

### Study Characteristics

**Figure 1:**
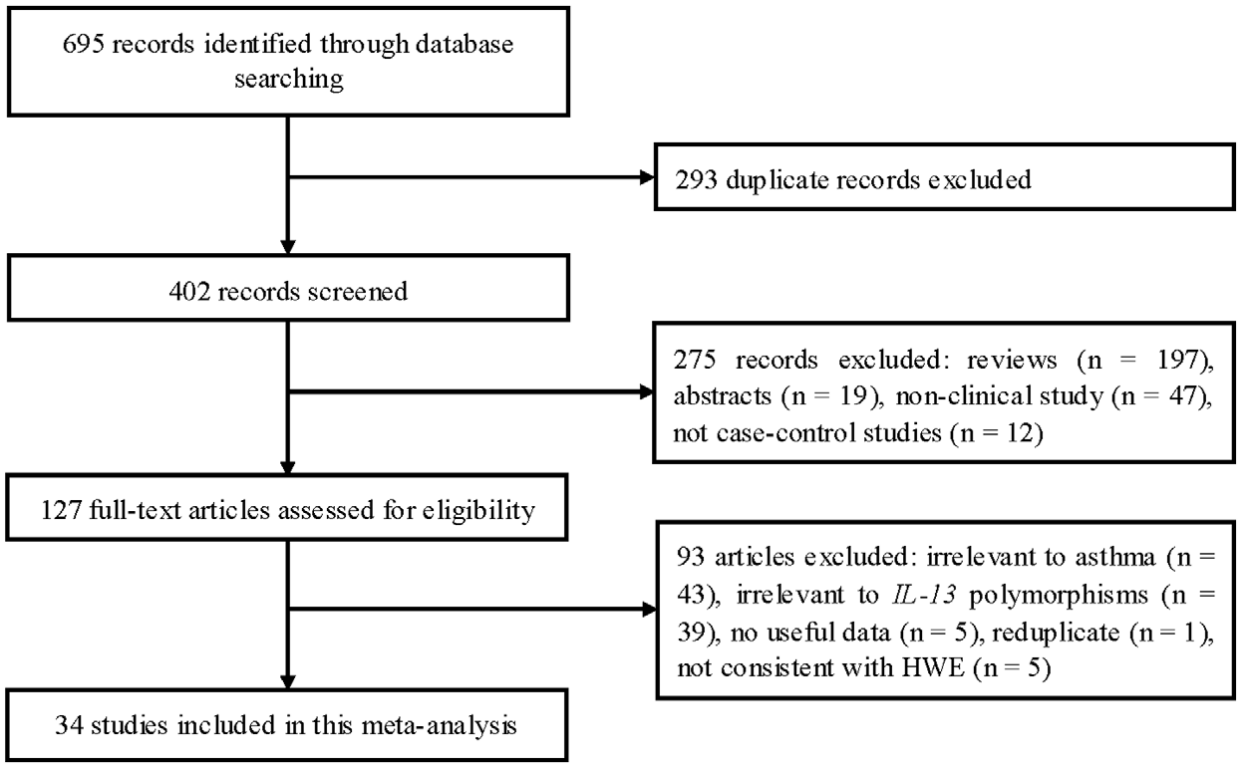
Flow of study identification, inclusion, and exclusion.

The flow chart in **Figure 1** summarizes this literature review process. In this current study, a total of 34 eligible studies met the inclusion criteria [7]–[40]. Four articles reported two cohorts [12], [14], [33], [38], and each cohort was considered as a case-control study. There were 22 studies on -1112C/T polymorphism and 31 studies on +2044A/G polymorphism. There were 18 studies performed using Asians, 13 studies using Caucasians, and 4 studies using African Americans. Ten studies were performed in adults and seventeen in children. Seven studies included only atopic asthma patients, six studies included both of atopic and non-atopic asthma patients but data for these patients could be separately extracted, and 19 studies did not report detailed information. Quality scores for the individual studies ranged from 5 to 12. The characteristics of each study included in this meta-analysis are presented in **Table 1**. Genotype frequencies and HWE examination results are listed in **Table 2**.

**Table 1:** Characteristics of the case-control studies included in meta-analysis.

First authors/references	Year	Country	Ethnicity	Age group	Atopic status	Case(n)	Control(n)	Genotypingmethod	Qualityscore
van der Pouw Kraan [7]	1999	Netherlands	Caucasian	NA	Atopic	101	107	PCR-OLA	5
Hakonarson [8]	2001	Iceland	Caucasian	Mixed	Atopic	94	94	PCR	10
Howard [9]	2001	Holland	Caucasian	Adults	Mixed*	171	119	Sequencing	9
Kauppi [10]	2001	Finland	Caucasian	NA	NA	163	132	PCR	7
Leung [11]	2001	China	Asian	Children	Mixed*	157	54	PCR-RFLP	9
Xi 1 [12]	2004	China	Asian	Adults	NA	45	46	PCR-RFLP	5
Xi 2 [12]	2004	China	Asian	Children	NA	43	31	PCR-RFLP	5
Wu [13]	2004	China	Asian	Mixed	NA	100	100	PCR-RFLP	7
Donfack 1 [14]	2005	USA	Caucasian	NA	Mixed*	126	205	DNAprint, LAS	9
Donfack 2 [14]	2005	USA	African American	NA	Mixed*	205	183	DNAprint, LAS	9
Moissidis [15]	2005	USA	African American	Mixed	NA	61	157	PCR-RFLP	5
Zhao [16]	2005	China	Asian	Children	NA	130	100	PCR-RFLP	7
Kabesch [17]	2006	Germany	Caucasian	Children	NA	73	773	PCR-RFLP	9
Battle [18]	2007	USA	African American	Mixed	NA	264	176	PCR-RFLP	11
Kang [19]	2007	Korea	Asian	Children	NA	374	242	PCR-RFLP	11
Chan [20]	2008	China	Asian	Children	Mixed	273	141	PCR-RFLP	7
Kim [21]	2008	Korea	Asian	Children	Mixed*	715	240	PCR-RFLP	10
Black [22]	2009	UK	Caucasian	Adults	NA	275	2453	Tetra primer PCR	11
Daley [23]	2009	Australia	Caucasian	Mixed	NA	644	751	Illumina	9
								Bead Array	
								System	
H Li [24]	2009	China	Asian	Children	NA	192	192	PCR-RFLP	8
Jiang [25]	2009	China	Asian	Mixed	NA	24	24	PCR-RFLP	7
Llanes [26]	2009	Spain	Caucasian	Adults	Atopic	109	50	PCR-RFLP	8
Wang [27]	2009	China	Asian	Children	Mixed	446	511	Taqman	8
Feng [28]	2009	China	Asian	Children	NA	45	43	PCR	6
Wang [29]	2009	China	Asian	Adults	NA	150	160	PCR-RFLP	6
Bottema [30]	2010	Netherlands	Caucasian	Adults	Atopic	115	92	MassARRAY	8
Dmitrieva-Zdorova [31]	2010	Russia	Caucasian	Adults	Atopic	283	227	MALDI-TOF mass-spectrometry	5
Palikhe [32]	2010	Korea	Asian	Adults	NA	463	430	SNAPshot	6
Undarmaa 1 [33]	2010	Japan	Asian	Children	Atopic	325	336	TaqMan-ASA	9
Undarmaa 2 [33]	2010	Japan	Asian	Adults	Atopic	367	676	TaqMan-ASA	9
Wu XH [34]	2010	China	Asian	Children	NA	252	227	PCR-RFLP	8
Yang LF [35]	2010	China	Asian	Children	NA	178	158	PCR-RFLP	5
DeWan [36]	2010	USA	Mixed	Children	Atopic	104	503	Affymetrix	11
								Genome-Wide Human	
								SNP Array 5.0, TaqMan	
Yang XX [37]	2011	China	Asian	Adults	Mixed*	193	204	MALDI-TOF mass-spectrometry	7
Baye 1 [38]	2011	USA	Caucasian	Children	NA	413	298	IGGAS	9
Baye 2 [38]	2011	USA	African American	Children	NA	315	51	IGGAS	9
Noguchi [39]	2011	Japan	Asian	Children	Mixed	938	2376	Illumina HumanHap550v3	12
								/610-Quad Genotyping BeadChip	
Munoz [40]	2012	Mexico	Caucasian	Children	NA	90	111	TaqMan	5

* Data for atopic or non-atopic asthma patients could be separately extracted.

PCR, polymerase chain reaction; OLA, oligonucleotide ligase assay; RFLP, restriction fragment length polymorphism; LAS, multiplex PCR and an immobilized linear array system; TaqMan-ASA, TaqMan allele-specific amplification method; IGGAS, Illumina GoldenGate Assay system; NA, not available.

**Table 2:** Distribution of *IL-13* genotype among patients and controls.

Studies		Asthma			Control		HWE
	11^a^	12^b^	22^c^	11	12	22	(*P* value)
−1112C/T							
van der Pouw Kraan	57	31	13	77	28	2	0.765
Howard	99	63	9	87	30	2	0.748
Wu	50	37	13	69	25	6	0.087
Donfack 1	72	42	12	126	71	8	0.607
Donfack 2	69	100	36	66	85	32	0.609
Moissidis	13	36	12	62	75	20	0.712
Kabesch	34	33	6	471	263	39	0.770
Battle	95	126	42	58	85	30	0.905
Kang	236	128	10	156	79	6	0.276
Kim	455	236	25	155	80	6	0.246
Black	158	98	7	1609	673	80	0.353
Daley	425	195	22	490	234	27	0.886
H Li	136	47	9	141	45	6	0.312
Wang	321	113	12	357	136	18	0.265
Bottema	67	43	5	65	23	4	0.301
Dmitrieva-Zdorova	149	116	18	117	94	16	0.623
Undarmaa 1	190	117	18	227	98	11	0.915
Undarmaa 2	230	121	16	459	196	21	0.989
Yang XX	144	43	6	148	50	6	0.484
Baye 1	243	148	22	187	98	13	0.972
Baye 2	115	151	49	18	25	8	0.889
Munoz	45	34	11	58	46	7	0.594
+2044A/G							
Hakonarson	3	25	66	3	27	64	0.941
Howard	11	52	89	9	44	67	0.637
Kauppi	17	82	64	19	51	62	0.119
Leung	29	74	54	7	26	21	0.812
Xi 1	6	24	15	3	20	23	0.624
Xi 2	8	25	10	2	13	16	0.765
Donfack 1	7	41	79	5	73	127	0.141
Donfack 2	6	67	132	4	53	126	0.564
Zhao	52	60	18	50	42	8	0.842
Battle	9	81	171	5	52	117	0.787
Kang	48	166	160	28	100	101	0.673
Chan	43	136	94	17	70	54	0.431
Kim	90	318	301	28	100	99	0.724
Black	11	98	166	76	657	1729	0.161
Daley	22	196	426	21	209	520	0.999
Jiang	2	2	20	1	5	18	0.422
Llanes	2	38	68	4	54	87	0.194
Wang	49	194	203	59	234	212	0.646
Feng	10	19	17	3	10	30	0.128
Bottema	6	51	57	3	24	62	0.721
Dmitrieva-Zdorova	23	116	144	17	85	125	0.630
Palikhe	56	200	207	50	174	206	0.158
Undarmaa 1	36	144	145	34	149	156	0.856
Undarmaa 2	39	162	166	65	289	322	0.989
Wu XH	36	111	105	18	84	125	0.465
Yang LF	47	60	71	19	66	73	0.497
DeWan	5	34	65	23	171	309	0.915
Baye 1	26	157	230	14	101	183	0.989
Baye 2	8	87	220	1	14	36	0.787
Noguchi	113	438	387	232	1033	1111	0.718
Munoz	21	52	17	23	65	23	0.071

a CC or AA;

b CT or AG;

c TT or GG.

HWE, Hardy-Weinberg equilibrium.

### Quantitative Data Synthesis

#### The *IL-13* −1112C/T polymorphism

**Figure 2:**
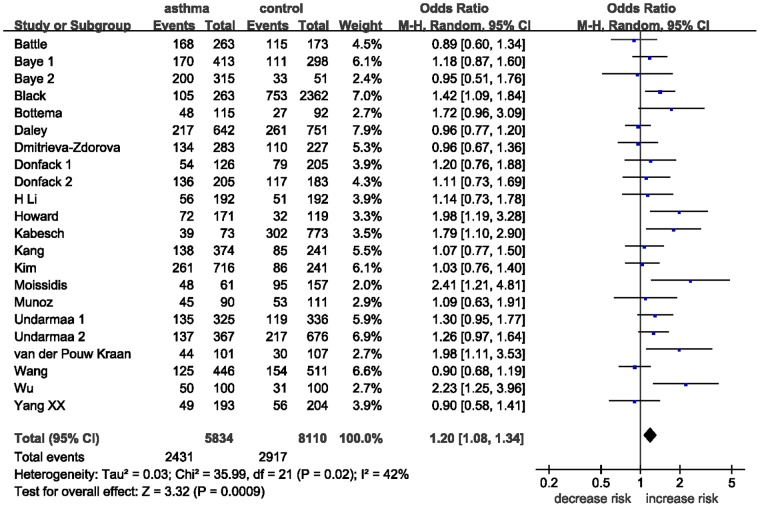
Meta-analysis for the association between asthma risk and the *IL-13* −1112C/T polymorphism.

Twenty-two studies determined the association between −1112C/T polymorphism and asthma. The sample sizes for case and control groups were 5834 and 8110, respectively. The estimated OR1, OR2 and OR3 were 1.32 (*P* = 0.002), 1.17 (*P* = 0.002), and 1.12 (*P* = 0.21) (**Table 3**). These estimates suggested a dominant genetic model, therefore TT and TC were combined and compared with CC. The pooled OR was 1.20 (95% CI 1.08–1.34, *P* = 0.0009) (**Figure 2**). There was moderate heterogeneity (*I^2^* = 42%, *P = *0.02). In the stratified analysis by ethnicity, a statistically significant association was found for studies with Caucasians (OR = 1.30, 95% CI 1.09–1.55, *P = *0.003). However, no significant association was observed in Asians and African Americans (**Table 3**). In the subgroup analysis by atopic status, the *IL-13* −1112C/T polymorphism was significantly associated with risk of atopic asthma (OR = 1.25, 95% CI 1.07–1.45, *P = *0.004) but not with non-atopic asthma risk (OR = 1.28, 95% CI 0.97–1.68, *P = *0.08). Of note, heterogeneity was significantly decreased in atopic asthma subgroup and non-atopic asthma subgroup (*I^2^* = 22%, *P = *0.24, and *I^2^* = 0%, *P = *0.86, respectively).

**Table 3:** Determination of the genetic effects of *IL-13* polymorphisms on asthma and subgroup analyses.

Polymorphisms	Study	Sample size		No. of studies	Test of association			Model		Heterogeneity		
		case	control		OR (95% CI)	*Z*	*P* Value			*χ* ^2^	*P* Value	*I* ^2^ (%)
−1112C/T												
TT vs. CC	Overall	3776	5571	22	1.32 (1.11–1.58)	3.17	0.002		R	27.19	0.16	23.0
TC vs. CC	Overall	5461	7742	22	1.17 (1.06–1.30)	3.06	0.002		R	30.35	0.09	31.0
TT vs. TC	Overall	2431	2907	22	1.12 (0.94–1.34)	1.26	0.21		R	14.98	0.82	0.0
TT+TC vs. CC	Overall	5834	8110	22	1.20 (1.08–1.34)	3.32	0.0009		R	35.99	0.02	42.0
TT+TC vs. CC	Asian	2713	2501	8	1.13 (0.97–1.32)	1.16	0.11		R	10.55	0.16	34.0
TT+TC vs. CC	Caucasian	2277	5045	10	1.30 (1.09–1.55)	2.95	0.003		R	17.87	0.04	50.0
TT+TC vs. CC	African	844	564	4	1.16 (0.80–1.67)	0.77	0.44		R	6.21	0.10	52.0
	American											
TT+TC vs. CC	Atopic	2198	2390	10	1.25 (1.07–1.45)	2.90	0.004		R	11.60	0.24	22.0
TT+TC vs. CC	Non-atopic	297	952	5	1.28 (0.97–1.68)	1.73	0.08		R	1.29	0.86	0.0
+2044A/G												
AA vs. GG	Overall	4808	7055	31	1.28 (1.13–1.46)	3.81	0.0001		R	32.41	0.35	7.0
AG vs. GG	Overall	7277	10304	31	1.15 (1.06–1.25)	3.38	0.0007		R	37.69	0.16	20.0
AA vs. AG	Overall	4151	4935	31	1.11 (0.99–1.25)	1.78	0.08		R	20.39	0.91	0.0
AA+AG vs. GG	Overall	8118	11147	31	1.18 (1.08–1.28)	3.78	0.0002		R	44.30	0.04	32.0
AA+AG vs. GG	Asian	4770	5603	16	1.19 (1.04–1.36)	2.49	0.01		R	29.20	0.02	49.0
AA+AG vs. GG	Caucasian	2463	4633	11	1.22 (1.06–1.40)	2.82	0.005		R	13.25	0.21	25.0
AA+AG vs. GG	African	781	408	3	1.13 (0.86–1.47)	0.88	0.38		R	0.25	0.88	0.0
	American											
AA+AG vs. GG	Atopic	2486	2827	12	1.12 (1.00–1.26)	1.92	0.05		R	10.60	0.48	0.0
AA+AG vs. GG	Non-atopic	259	789	5	0.87 (0.60–1.27)	0.72	0.47		R	5.42	0.25	26.0

vs., versus; R, random-effects model.

**Figure 3:**
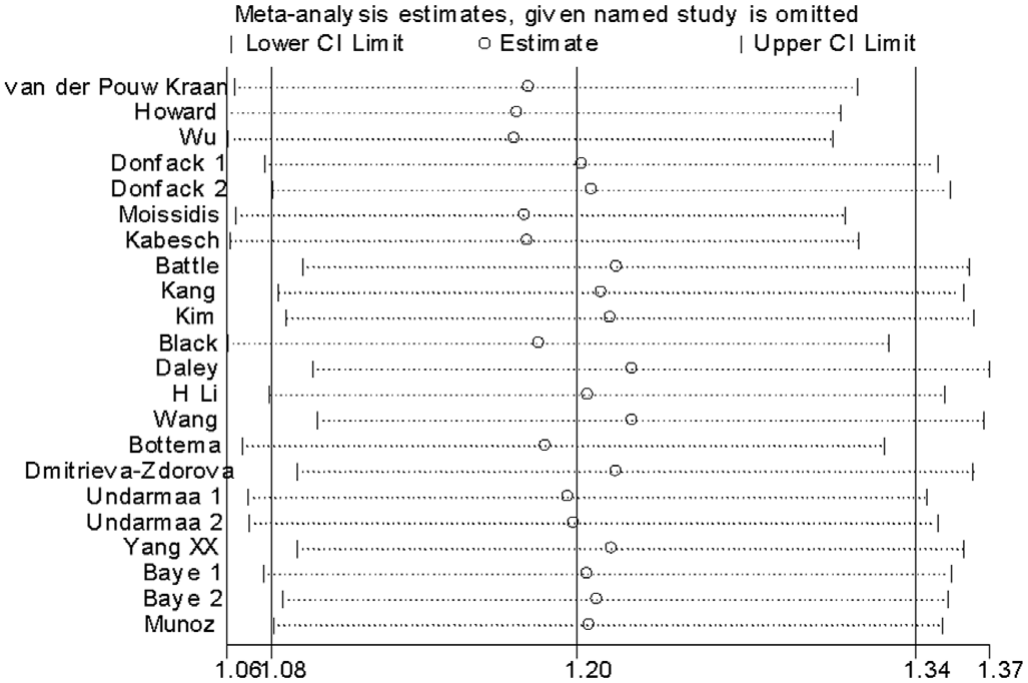
One-way sensitivity analysis for the *IL-13* −1112C/T polymorphism with asthma risk.

**Figure 4:**
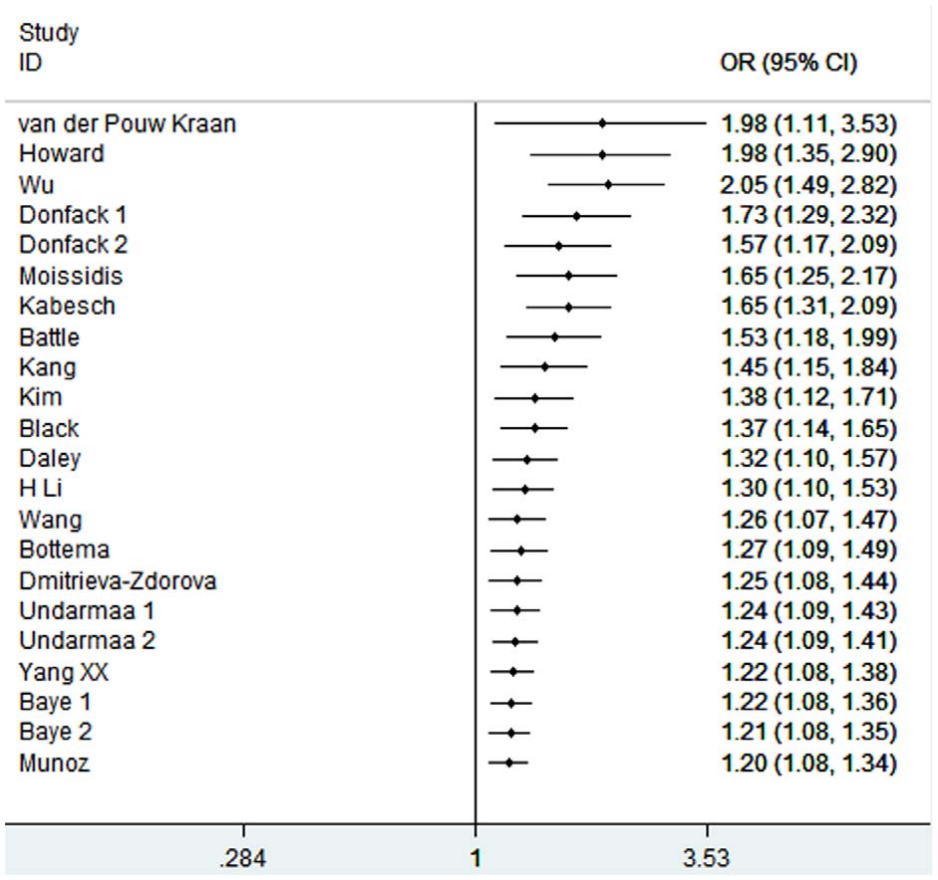
Cumulative meta-analysis of associations between the *IL-13* −1112C/T polymorphism and asthma risk.

**Figure 5:**
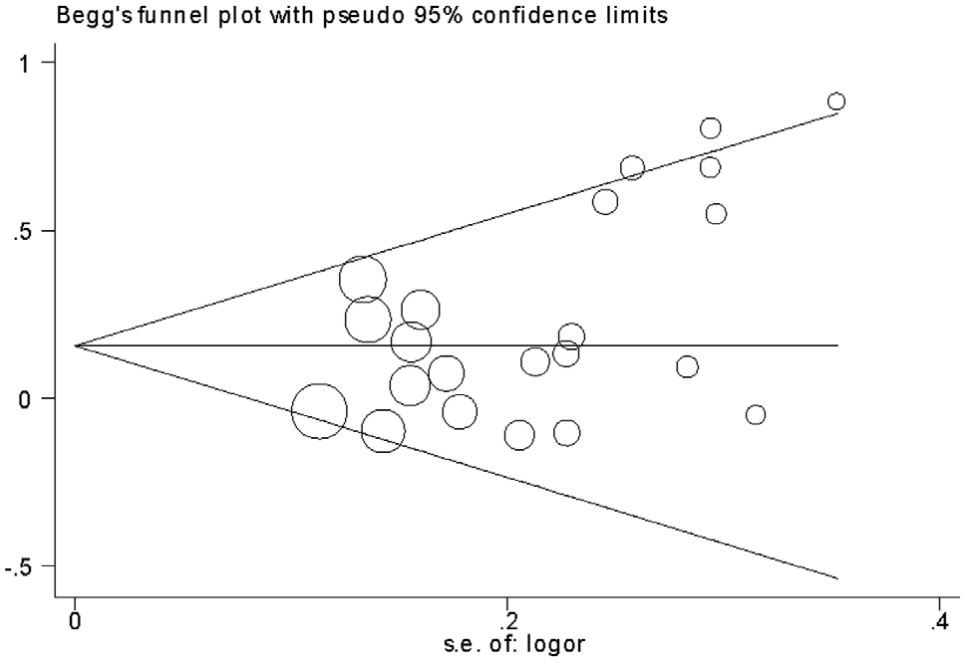
Funnel plot for asthma risk and the *IL-13* −1112C/T polymorphism.

We conducted one-way sensitivity analysis to evaluate the stability of the meta-analysis. As shown in **Figure 3**, the statistical significance of the results was not altered when any single study was omitted. Cumulative meta-analyses of *IL-13* −1112C/T polymorphism association were also conducted. The inclination toward significant association with asthma risk was found (**Figure 4**). The funnel plot was seemed symmetrical (**Figure 5**). However, Egger’s test indicated significant publication bias (*P = *0.021).

#### The *IL-13*+2044A/G polymorphism

**Figure 6:**
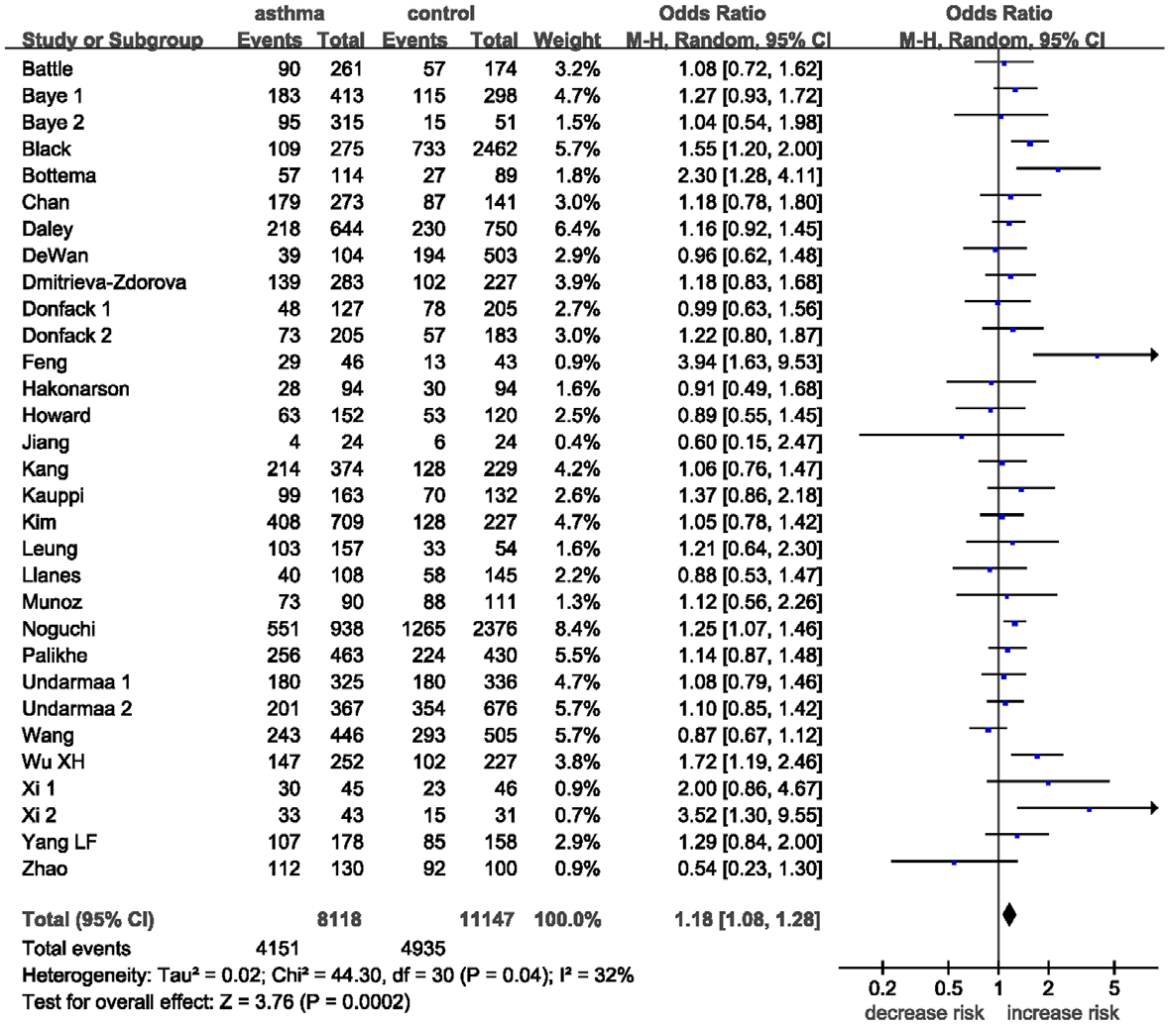
Meta-analysis for the association between asthma risk and the *IL-13*+2044A/G polymorphism.

Thirty-one case-control studies identified an association between *IL-13*+2044A/G polymorphism and asthma risk. A total of 8118 cases and 11147 controls were included in this meta-analysis. The estimated OR1, OR2 and OR3 were 1.28 (*P* = 0.0001), 1.15 (*P* = 0.0007), and 1.11 (*P* = 0.08), respectively (**Table 3**). Thus, these estimates suggested a dominant genetic model, therefore AA and AG were combined and compared with GG. The pooled OR was 1.18 (95% CI 1.08–1.28, *P* = 0.0002) (**Figure 6**). Moderate heterogeneity (*I^2^* = 32%, *P = *0.04) was found. Subgroup analysis was performed by ethnicity. Statistically significant findings were witnessed in Asians (OR = 1.19, 95% CI 1.04–1.36, *P = *0.01) and Caucasians (OR = 1.22, 95% CI 1.06–1.40, *P = *0.005) but not in African Americans. In terms of atopic status, borderline yet significant increased asthma risk was found among atopic asthma patients (OR = 1.12, 95% CI 1.00–1.26, *P = *0.05), but no statistically significant finding was found among non-atopic asthma patients (OR = 0.87, 95% CI 0.60–1.27, *P = *0.47). Again, significant decreased heterogeneity was found in atopic asthma subgroup and non-atopic asthma subgroup (*I^2^* = 0%, *P = *0.48, and *I^2^* = 26%, *P = *0.25, respectively).

**Figure 7:**
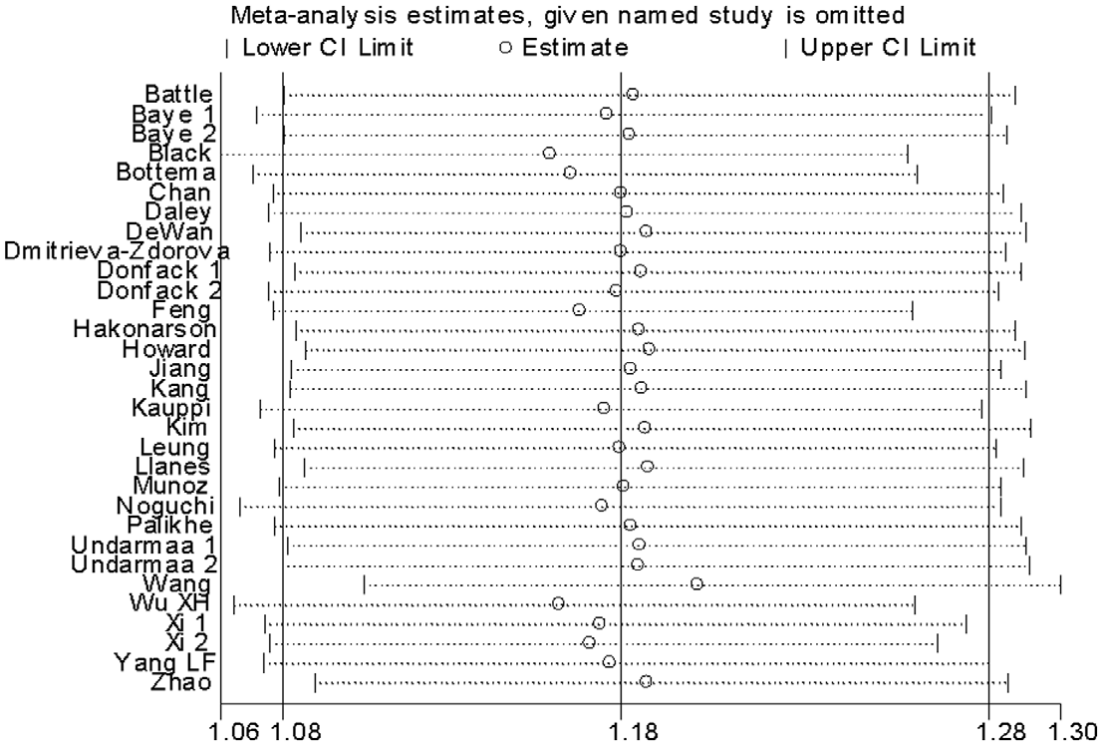
One-way sensitivity analysis for the *IL-13*+2044A/G polymorphism with asthma risk.

**Figure 8:**
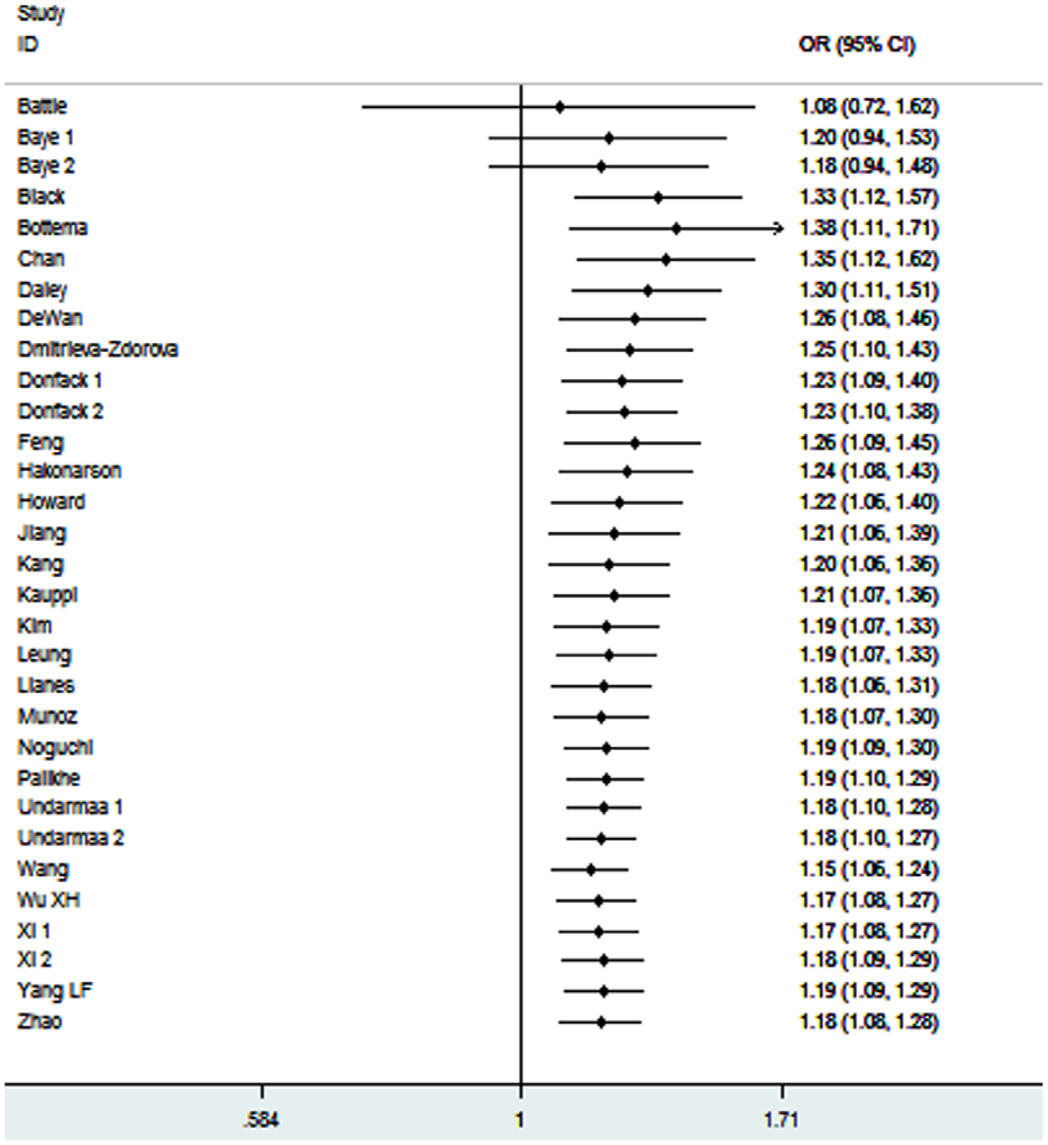
Cumulative meta-analysis of associations between the *IL-13*+2044A/G polymorphism and asthma risk.

**Figure 9:**
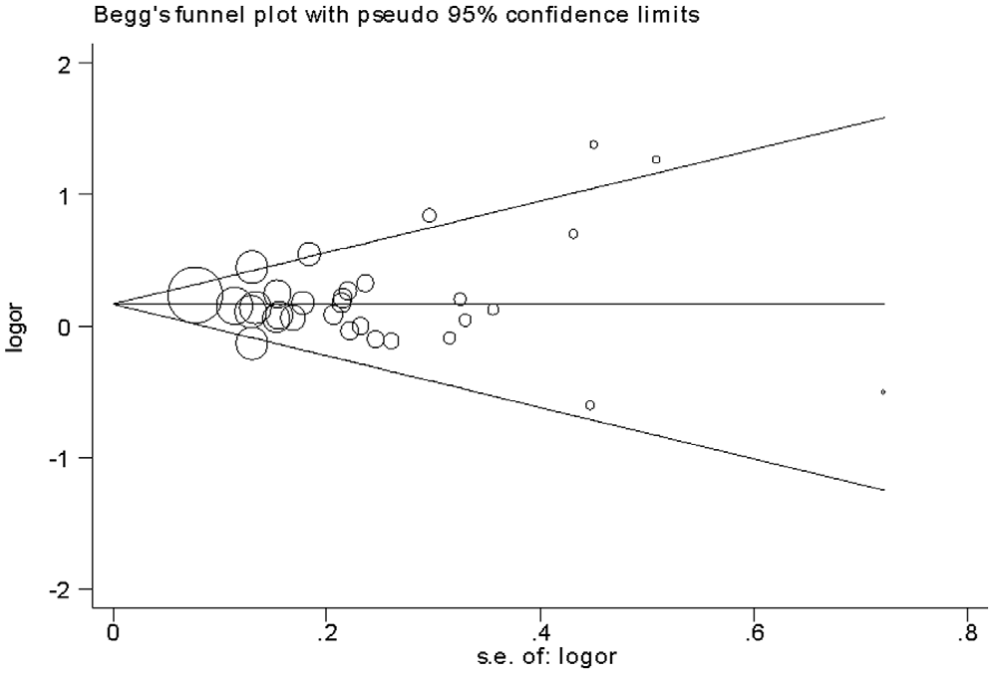
Funnel plot for asthma risk and the *IL-13*+2044A/G polymorphism.

In the one-way sensitivity analysis, there was little modification of the estimates after exclusion of individual study (**Figure 7**). Cumulative meta-analysis showed that the evidence was consistent over time (**Figure 8**). The shape of the funnel plots seemed symmetrical in the dominant genetic model (**Figure 9**). Egger’s test was used to provide statistical evidence of funnel plot symmetry. The result did not show any evidence of publication bias (*P = *0.684).

## Discussion

Hallmarks of asthma include airway inflammation predominated by eosinophils, mucus hyperproduction, and airway hyperresponsiveness (AHR) [49]. A considerable weight of evidence supporting a role for IL-13 in asthma was derived from animal models. For instance, previous studies showed that acute administration of IL-13 itself was sufficient to recapitulate eosinophilic inflammation in nonimmunized mice or recombination-activating gene-deficient mice [50], [51]. In addition, blockade of IL-13 alone in vivo through *IL-13* gene targeting in mice prevented and reversed established mucus cell changes, suggesting a key role of IL-13 in mucus hyperproduction [52], [53] Furthermore, AHR can be induced by IL-13 overexpression and blockade of IL-13 by the soluble receptor-Fc fusion protein abrogated allergen-induced AHR [54]. Taken together, these results suggested that IL-13 was a critical cytokine in the development of asthma. *IL-13* was one of the most studied of the candidate genes for asthma. *IL-13* −1112C/T polymorphism led to increased *IL-13* transcription in polarized TH2 cells and enhanced IL-13 secretion by mitogen-stimulated mononuclear cells [55]. Moreover, Arima et al. [56] indicated that the *IL-13*+2044A/G polymorphism may be a functional variant. Studies demonstrated that the AA genotype resulted in decreased affinity of IL-13 for IL-13Rα2 and increased expression of IL-13 [56]. Thus, it is biologically plausible that these two polymorphisms could influence the susceptibility to asthma.

In the present meta-analysis, we explored the association between the *IL-13* −1112C/T and +2044A/G polymorphisms and asthma risk, including 34 eligible case-control studies. For *IL-13* −1112C/T polymorphism, 5834 cases and 8110 controls were included. We found that individuals with the −1112T allele (TT or TC) showed an increased risk of asthma in the overall population. The results from meta-analysis showed that carriers of the TT or TC genotype had 20% increased asthma risk compared to those individuals with the CC carriers. In the stratified analysis by ethnicity, the significant association was observed in Caucasians, but not in Asians and African Americans. It is possible that different genetic backgrounds may account for these differences. However, there were only four studies using African Americans. Thus, the positive association between African Americans and asthma could not be ruled out because studies with small sample size may have insufficient statistical power to detect a slight effect. In addition, significant heterogeneity (*I^2^* = 52%, *P = *0.10) may also distort the result. In the subgroup analysis by atopic status, we found *IL-13* −1112C/T polymorphism exhibited increased atopic asthma risk. For *IL-13*+2044A/G polymorphism, 8118 cases and 11147 controls were included. There was a significant association between this polymorphism and asthma risk. When subgroup analysis was performed according to ethnicity, significant associations were showed in Asians and Caucasians, but not in African Americans. Only three studies were performed with African Americans, thus the positive association still can not be excluded. The subgroup analysis based on atopic status found that *IL-13*+2044A/G polymorphism was marginally associated with allergic asthma risk. Taken together, these results suggested that *IL-13* polymorphisms may play a role in the etiology of allergic asthma.

A recent meta-analysis performed by Yang et al. [41] found +2044A/G polymorphism was associated asthma risk in Asians but not in Caucasians. Another meta-analysis conducted by Cui et al. [42] showed this polymorphism was more pronounced among Caucasians but not among Asians. Results from our study were inconsistent with these meta-analyses. We found significant associations in both Asians and Caucasians. There are several potential explanations for the different results. First, different inclusion and exclusion criteria were used in these two meta-analyses [41], [42]. For example, Cui et al. [42] only included English papers. However, Yang et al. [41] included articles published in English and Chinese. Thus, although these two meta-analyses were published in the same year, it was possible that different results may be observed. Second, different numbers of subjects were included in the two meta-analyses [41], [42]. For +2044A/G polymorphism, Cui et al. [42] included 8439 subjects in their study, while Yang et al. [41] only included 5695 subjects in their meta-analysis. Third, we noted that three studies (n = 806) performed using Caucasians and nine studies (n = 4241) performed using Asians were included in Yang’s study [41]. Moreover, six studies (n = 4202) conducted in Caucasians and five studies (n = 3673) conducted in Asians were included in Cui’s study [42]. Therefore, different statistical power might be another reason for the discrepant results. For +2044A/G polymorphism, our meta-analysis included eleven case-control studies (n = 7096) in Caucasians and sixteen case-control studies (n = 10373) in Asians, thus our study was more conclusive and more powerful. Additionally, our study had some advantages. First, we attempted to find as many publications as we could by means of various searching approaches. Second, it is the first time studying the atopic specificity and *IL-13* polymorphisms interactions. Third, the methodological issues for meta-analysis, such as, one-way sensitivity analysis and cumulative meta-analysis were well investigated.

Results from one-way sensitivity analysis and cumulative meta-analysis suggested high stability and reliability of our results. Besides, we had to mention the importance of heterogeneity and publication bias, which might influence the results of meta-analysis. In our study, moderate heterogeneity was observed for the *IL-13* −1112C/T and +2044A/G polymorphisms. We used subgroup analysis to explore the sources of heterogeneity. After subgroup analysis by atopic status, the heterogeneity was effectively decreased and disappeared. Therefore, the main source of heterogeneity was from atopic status. Moreover, funnel plots and Egger’s tests were used to find potential publication bias. The results indicated that there was significant publication bias for *IL-13* −1112C/T polymorphism. Thus, our results should be interpreted with caution and more studies are still needed to evaluate the effect of *IL-13* −1112C/T polymorphism on asthma risk.

Several limitations need to be addressed. First, the numbers of published studies were not sufficient for a comprehensive analysis, particularly for African Americans. Second, our results were based on unadjusted estimates. Lacking of the original data of the eligible studies limited the evaluation of the effects of the gene-gene and gene-environment interactions in asthma. Third, Vercelli [57] suggested that *IL-13* −1112C/T and +2044A/G were in high linkage disequilibrium. However, we did not carry out haplotype analysis due to limited data.

In conclusion, this meta-analysis suggested that *IL-13* −1112C/T and +2044A/G polymorphisms may be associated with the risk of asthma. Well-designed studies with larger sample size and more ethnic groups should be considered to further confirm these associations, especially in African Americans.
